# Spectrophotometric Estimation of Ketotifen Fumarate from Tablet Formulations

**DOI:** 10.4103/0250-474X.51964

**Published:** 2009

**Authors:** I. Singhvi, D. Sachdeva

**Affiliations:** Pacific College of Pharmacy, Pacific Hills, Pratap Nagar, Air-port Road, Udaipur-313 001, India; 1 Sanjeevan College of Pharmacy, Dausa-303 303, India

**Keywords:** Spectrophotometric, ketotifen fumarate, 2-nitroso- napthol-4-sulphonic acid and rhodizonic acid

## Abstract

Two simple and sensitive visible spectrophotometric methods have been developed for the quantitative estimation of ketotifen fumarate from its tablet formulation. The developed methods are based on formation of chloroform extractable colored complex of with 2-nitroso- napthol-4- sulphonic acid and rhodizonic acid. The extracted complex of drug with 2-nitroso- napthol-4- sulphonic acid (method-I), showed absorbance maxima at 436.5 nm and with rhodizonic acid (method-II), showed absorbance maxima at 489.5 nm. The linearity range for both the developed methods was observed in the concentration range of 50-250 µg/ml of drug. Results of analysis for both the developed methods were validated statistically and by recovery studies.

Ketotifen fumarate, 4,9-dihydro-4-(1-methyl-4-piperidinylidene)-10-H-benzo[4,5]cycloheptal-[1,2-b]thiophen-10-one fumarate, is used as an antiasthamatic agent[1]. Few analytical methods for estimation of ketotifen fumarate including GC[2,3], HPLC[4–6] and spectrophotmetric[7–9] are reported.

A Jasco UV/Vis double beam spectrophotometer (model 7800) with 1 cm matched quartz cells was used for spectral measurement. All the chemical used were of analytical grade, a 1% solution of 2-nitroso-napthol-4-sulphonic acid (Thomas Baker, Mumbai, India) and 2% solution of rhodizonic acid (Thomas Baker, Mumbai, India) were prepared in distilled water and extracted several times with chloroform so as to remove chloroform soluble impurities. Standard drug solution of ketotifen fumarate (500 µg/ml) was prepared in distilled water. The tablet sample of ketotifen fumarate was procured from the local pharmacy.

In a series of 10 ml volumetric flasks aliquots of standard drug solution of ketotifen fumarate in distilled water were transferred and diluted with the same so as to give several dilutions in the concentration range of 50-250 µg/ml of drug. For the method I, to each dilution (5 ml) taken in a separating funnel, 5 ml of 2-nitroso-napthol-4-sulphonic acid solution was added and shaken for 10 min for the formation of colored complex. The colored complex was extracted with 5, 3, and 2 ml portions of chloroform, the volume of combined chloroform layer was made up to 10 ml and absorbance was measured at 436.5 nm against a reagent blank. A calibration curve was prepared by plotting concentration versus absorbance.

For the method II, to each dilution (5 ml) taken in a separating funnel, 5 ml of rhodizonic acid solution was added, shaken and allowed to stand for 10 min for the formation of colored complex. The colored complex was extracted with 5, 3, and 2 ml portions of chloroform, the volume of combined chloroform layer was made up to 10 ml and absorbance was measured at 489.5 nm against a reagent blank. A calibration curve was prepared by plotting concentration versus absorbance.

For analysis of formulation, twenty tablets of ketotifen fumarate were accurately weighed and average weight per tablet was determined. The tablets were powdered and powder equivalent to 10 mg of ketotifen fumarate was accurately weighed and extracted four times with 20 ml portions of distilled water, the combined extract was filtered through Whatman filter paper No. 41 in to 100 ml volumetric flask. The residue was washed with distilled water and the washing was added to the filtrate, final volume of filtrate was made up to the mark with distilled water. Filtrate (10 ml) was treated as per the respective procedure for the calibration curve and absorbance was measured at 436.5 nm (method-I) and 489.5 nm (method-II), the amount of drug present in sample was computed from respective calibration curve.

Analysis for both the developed methods was repeated five times for three different batches of tablet formulations. Results of analysis are reported in Table 1. Recovery studies were carried out for both the developed methods by addition of known quantity of pure drug solution to pre analyzed tablet sample solution at three different concentration level. The result of recovery studies is reported in Table 1.

**TABLE 1:** ANALYSIS OF TABLET FORMULATION

Method	Formulation	Label Claim mg/tab	% of label claim estimated*	Standard deviation	% Recovery**	Standard deviation
I	Tablet I	01	99.63	0.685	99.06	0.653
Nitrso Napthol	Tablet II	01	98.78	0.794	99.65	0.893
	Tablet III	01	99.01	0.589	98.98	0.624
II	Tablet I	01	98.10	0.508	100.70	0.543
Rhodizonic acid	Tablet II	01	98.90	0.789	99.54	0.872
	Tablet III	01	99.00	0.812	99.84	0.842

* Average of five determinations.

** Average of determination at three different concentration level

The proposed spectrophotometric methods for determination of ketotifen fumarate from tablet formulations are based on formation of chloroform extractable colored complex of drug with 2-nitroso-napthol-4-sulphonic acid and rhodizonic acid. The results of analysis for both the developed methods were close to 100% and standard deviation was satisfactorily low indicating accuracy and reproducibility of the methods. Recovery studies were satisfactory which shows that there is no interference of excipients. The developed methods were found to be simple, rapid, accurate and can be used for routine analysis of drug from tablet formulations.
